# Quality of life: the perspective of neurodivergent university students

**DOI:** 10.1192/j.eurpsy.2025.1647

**Published:** 2025-08-26

**Authors:** I. S. Tsalamatas, M. E. N. Santos, D. Cardilli-Dias, D. R. Molini-Avejonas

**Affiliations:** 1 Department of Physiotherapy, Speech Therapy and Occupational Therapy, Faculty of Medicine of the University of São Paulo, São Paulo, Brazil

## Abstract

**Introduction:**

Neurodevelopmental disorders are prevalent worldwide, with an increase in diagnoses in recent years (Faraone et al. Neurosci Biobehav Rev. 2021; 789-818; Russel et al. J Child Psychol Psychiatry 2022; 674-682). Individuals diagnosed with conditions such as Autism Spectrum Disorder (ASD), Attention Deficit Hyperactivity Disorder (ADHD), Specific Learning Disorder (SLD) and Language Development Disorder (DLD) are considered neurodivergent and constitute the so-called neurominorities (Doyle, N. British medical bulletin 2020; 108-125). Studies have shown that adults with ADHD and ASD have lower scores when assessed for quality of life, compared to neurotypicals (Pinho et al, J Atten Disord 2019; 1736-1745; Sáez-Suanes & Álvarez-Couto, Rev J Autism Dev Disord 2022; 307-319).

**Objectives:**

The present study aims to describe the quality of life of neurodivergent students.

**Methods:**

The research was cross-sectional, prospective and quantitative. The project was approved by the Research Ethics Committee. A total of 79 neurodivergent university students from public and private universities in the State of São Paulo participated in the research. The study was carried out remotely through the Google Forms platform with application of the TCLE and WHOQOL-DIS instruments.

**Results:**

It was observed that 30% of the participants had a diagnosis of ASD, while 48% had ADHD, 8% had ADD, 14% had ASD with ADHD and none had a diagnosis of DLD or SLD. It is worth mentioning that in the questions about quality of life and health, 18% were dissatisfied. Regarding the ability to perform tasks, 13% reported that physical pain prevented them and 70% reported needing medical treatment. Regarding levels of personal satisfaction, 37% said they were dissatisfied with their sleep, 20% were not satisfied as a person and 28% scored completely dissatisfied with access to health services. Regarding well-being and neurodivergence, 30% of the participants were completely unhappy and 15% stated that their limitation had a negative effect on their life. Regarding autonomy, belonging, and self-perception, 23% reported total dissatisfaction with their communication skills and 41% stated that they were completely dissatisfied with their involvement in social activities. Finally, regarding strengths and weaknesses, 32% of the subjects reported not being at all satisfied with their work opportunities, 29% were in the same situation regarding the adaptations of the environment to their limitations, and 14% reported dissatisfaction with study opportunities.

**Conclusions:**

This study was able to analyze the perspective on the quality of life of neurodivergent university students. However, due to the various biases that involve this population, it is necessary to seek broader answers, looking at the national scope to provide a better understanding of quality of life, including in the academic environment.

**Disclosure of Interest:**

None Declared

